# Infective endocarditis in intravenous drug abusers: clinical challenges emerging from a single-centre experience

**DOI:** 10.1186/s12879-021-06697-1

**Published:** 2021-09-27

**Authors:** Valentina Scheggi, Stefano Del Pace, Nicole Ceschia, Francesco Vanni, Irene Merilli, Edoardo Sottili, Leonardo Salcuni, Nicola Zoppetti, Bruno Alterini, Alfredo Cerillo, Niccolò Marchionni, Pier Luigi Stefàno

**Affiliations:** 1 Division of Cardiovascular and Perioperative Medicine, Cardiothoracovascular Department, Azienda Ospedaliero-Universitaria Careggi and University of Florence, Largo Brambilla 3, 50134 Florence, Italy; 2 Division of General Cardiology, Cardiothoracovascular Department, Azienda Ospedaliero-Universitaria Careggi and University of Florence, Largo Brambilla 3, 50134 Florence, Italy; 3 Division of Cardiac Surgery, Cardiothoracovascular Department, Azienda Ospedaliero-Universitaria Careggi and University of Florence, Largo Brambilla 3, 50134 Florence, Italy; 4 Institute of Applied Physics “Nello Carrara” (IFAC), National Research Council, Sesto Fiorentino, Italy; 5 Department of Experimental and Clinical Medicine, University of Florence, Florence, Italy

**Keywords:** Intravenous drug abuse, Infective endocarditis, Prognosis, Cardiac surgery

## Abstract

**Background:**

Intravenous drug abuse (IDA) is a known risk factor for infective endocarditis (IE) and is associated with frequent relapses, but its prognostic impact is still debated. The potential futility of surgery in this population is a further issue under discussion. We aimed to describe the clinical characteristics, the therapeutic strategy, and the prognosis associated with IDA in IE.

**Methods:**

We retrospectively analysed 440 patients admitted to a single surgical centre for definite active IE from January 2012 to December 2020.

**Results:**

Patients reporting IDA (N = 54; 12.2%) were significantly younger (p < 0.001) and presented fewer comorbidities (p < 0.001). IDA was associated with a higher proportion of relapses (27.8 vs. 3.3%, p < 0.001) and, at multivariable analysis, was an independent predictor of long-term mortality (HR 2.3, 95%CI 1.1–4.7, p = 0.015). We did not register multiple relapses in non-IDA patients. Among IDA patients, we observed 1 relapse after discharge in 9 patients, 2 relapses in 5 patients and 3 relapses in 1 patient. In IDA patients, neither clinical and laboratory variables nor the occurrence of even multiple relapses emerged as indicators of an adverse risk–benefit ratio of surgery in patients with surgical indication.

**Conclusions:**

IE secondary to IDA affects younger patients than those with IE not associated with IDA. Probably due to this difference, IE secondary to IDA is not associated with significantly higher mortality, whereas the negative, long-term prognostic impact of IDA emerges in multivariate analysis. Considering the good prognosis of patients with uncomplicated IE treated medically, surgery should be reserved to patients with a strict- guidelines-based indication. However, since there are no clear predictors of an unfavourable risk–benefit ratio of surgery in patients with surgical indication, all patients with a complicated IE should be operated, irrespective of a history of IDA.

## Introduction

Intravenous drug abuse (IDA) is a known risk factor for infective endocarditis (IE) and is associated with frequent relapses [1], but its prognosis, and the potential high risk–benefit ratio of surgery in the population of drug abusers with IF, are still debated issues.

Compared to the general population, IDA is associated with an up to 100-fold increased risk of IE through several mechanisms, including endothelial injury from injected particulate matter, direct injection of contaminated material, and drug-associated vasospasm leading to intimal damage and thrombus formation [1]. As a consequence of its pathophysiology, IDA-associated IE is more commonly right-sided [2]. Beyond these known characteristics, however, literature on this topic is substantially limited, and only a few studies have examined the outcome after acute hospitalization [2–6]. The best therapeutic strategy in IDA-associated IE, whether medical or surgical, remains unclear. In particular, given the high risk of relapses due to continued drug abuse, the decision to re-operate the same individual for a relapsed IE is sometimes challenging [2]. This study aimed to describe the clinical characteristics, the therapeutic strategy, and the prognosis of IDA-associated IE.

## Methods

### Patient selection

We retrospectively included in the analysis 440 patients with definite and active, non-device related IE, referred to a single surgical centre from January 2012 to December 2020. Device-related IE was defined as an infection involving cardiac devices other than prosthetic valves. Demographic and clinical data for analysis were retrieved from electronic hospital charts. The local Ethics Committee approved the study (authorization n 12113_oss) and, given the retrospective and non-interventional nature of the study, granted a waiver of informed consent. The diagnostic work-up and treatment strategies adhered to the current international IE guidelines [7]. At least three sets of blood cultures were collected and transesophageal (TE) echocardiography was performed for diagnostic confirmation in all patients. All patients received appropriate empirical and, when feasible, targeted antimicrobial therapy.

### Follow-up

The follow-up duration was calculated from IE diagnosis and updated to March 2021 through a structured phone interview or authorities’ registries in case of non-response. The mean follow-up was 3.0 ± 1.6 years in the whole study population.

### Study outcome

All-cause mortality and IE relapse rate were the primary and secondary study outcomes, respectively.

### Statistical analysis

Descriptive statistics are reported as mean ± standard deviation (SD) or median ± interquartile range (IQR) for continuous variables with normal and non-normal distribution and as frequencies and percentages for categorical variables. Between-groups comparisons were assessed by Student’s t-test, Mann–Whitney U test, Kruskal–Wallis, or chi-square test, as appropriate. The univariate and multivariate associations with long-term survival were analysed by the Kaplan–Meier method and by stepwise Cox proportional hazards models. In these analyses, patients with IE relapses were considered only once. Multivariate analysis of mortality was adjusted for the treatment received: surgery, medical therapy only in cases with no indication to surgery, or exclusion from surgery despite indication for too severe clinical conditions or refusal, as previously described [8]. Gender, age, diabetes, chronic kidney disease (CKD: eGFR < 60 ml/min/1.73m^2^), the microbiologic agent involved, left ventricular ejection fraction, type of valve (native or prosthetic), double valve infection, paravalvular extension of infection, severe valvular dysfunction, brain embolism detected on admission, EuroSCORE II, presence of a pacemaker, and history of drug abuse, were initially entered into the multivariate model, and then backward deleted when redundant (p out > 0.10). All analyses were performed with SPSS software version 24.0 (IBM Corp., Armonk, New York) and R version 3.6.3. Statistical significance was set at a 2-sided p-value < 0.05.

## Results

The main demographic, clinical and echocardiographic data of patients with IE associated (N = 54; 12.2%) and non-associated (N = 386) with IDA are reported in Table 1.

**Table 1:** Demographic and clinical characteristics, echocardiographic parameters, treatment strategy and microbiologic yield of the 440 patients with infective endocarditis (IE), by history of intravenous drug abuse (IDA)

	Non-IDA-associated IE	IDA-associated IE	
	N = 386	N = 54	p
Age, years (median ± IQR)	71 (17)	41 (18.5)	0.001
Female gender [n (%)]	138 (35.7)	15 (27.8)	NS
BMI (median ± IQR)	24.3 (5.0)	23.0 (6.1)	NS
Renal failure [n (%)]	102 (26.4)	8 (14.8)	NS
Hypertension [n (%)]	246 (63.7)	5 (9.2)	0.001
Previous cancer [n (%)]	89 (23.0)	1 (1.8)	0.001
Diabetes [n (%)]	80 (20.7)	2 (3.7)	0.001
Dyslipidemia [n (%)]	122 (31.6)	2 (3.7)	0.001
Pacemaker [n (%)]	45 (11.6)	7 (12.9)	NS
Associated viral infections [n (%)]	19 (4.9)	45 (83.3)	0.001
First episode of IE [n (%)]	362 (93.7)	38 (70.3)	0.001
IE on native valve [n (%)]	230 (59.5)	39 (72.2)	NS
Double-valve IE [n (%)]	68 (17.6)	7 (12.9)	NS
Left-sided IE [n (%)]	375 (97.1)	32 (59.2)	0.001
Embolism [n (%)]	147 (38.1)	34 (63.0)	0.001
Spondylodiscitis [n (%)]	28 (7.2)	9 (16.6)	0.020
Brain embolism [n (%)]	84 (21.8)	12 (22.2)	NS
Paravalvular extension [n (%)]	88 (22.7)	13 (22.2)	NS
Severe valvular dysfunction [n (%)]	180 (46.6)	31 (57.1)	NS
Vegetation length, mm (median ± IQR)	9.0 (10.0)	13.0 (12.5)	0.008
Ejection Fraction, % (median ± IQR)	60.0 (11.0)	60.0 (10.5)	NS
TAPSE, mm (mean ± SD)	21.0 (6.0)	23.0 (9.5)	0.02
EUROSCORE2 (median ± IQR)	7.8 (15.2)	3.6 (4.2)	0.001
No surgical indication [n (%)]	42 (10.9)	8 (12.6)	NS
Excluded from surgery despite indication [n (%)]	43 (11.1)	1 (3.1)	NS
Surgery [n (%)]	301 (78.0)	45 (84.1)	NS
Early surgery [n (%)]	238 (79.1)	38 (81.1)	NS
Polymicrobic IE [n (%)]	19 (4.9)	6 (12.7)	NS
Streptococci [n (%)]	98 (25.3)	7 (11.0)	0.045
Staphylococcus aureus [n (%)]	49 (12.7)	32 (54.0)	0.001
Negative coagulase Staphylococci [n (%)]	59 (15.3)	3 (9.5)	NS
Enterococci [n (%)]	79 (20.5)	7 (14.2)	NS
Negative culture [n (%)]	79 (20.5)	3 (9.5)	0.008
Other [n (%)]	22 (5.7)	2 (7.9)	NS

*BMI* body mass index, *TAPSE* tricuspid annular plane systolic excursion. Associated viral infections: HIV (human immunodeficiency virus), HBV (hepatitis B virus) and/or HCV (hepatitis C virus)

A history of fever (body temperature > 37.5 °C) was present in 278 patients (63%), without significant differences between IDA and non-IDA patient. The incidence of IDA-associated IE did not change over the study period. IDA patients were younger (41 ± 18.5 versus 71 ± 17, median ± IQR, p < 0.001) and – with the exception of a higher proportion of viral infections, such as HIV, HBV or HCV (p = 0.001) – they had fewer comorbidities (p < 0.001): therefore, their EuroSCORE II was significantly lower (p = 0.001). Consistently with the known high rate of relapses [1, 12] in drug abusers, IE was the first episode only in 70.3% of IDA-associated cases, a proportion much lower than the 93.7% observed in the other group (p < 0.001). As previously reported [8], IE in drug abusers was more frequently associated with spondylodiscitis (p = 0.02). As a consequence of its pathophysiological mechanism, the infection involved less frequently left-sided valves in IDA than in non-IDA patients (59.2 vs 97.1%, p < 0.001) and, because of prevalent right-sided infections, endocarditic vegetations were longer in IDA-associated IE (p < 0.001). Compared to non-IDA patients, Staphylococcus aureus (p < 0.001) and Streptococci (p = 0.045) were isolated respectively significantly more and less frequently from blood cultures of IDA patients. Culture negative IE was less frequent in IDA patients (9 vs 20%, p = 0.008). Embolism was present on admission in a higher proportion of IDA patients (p < 0.001), a finding consistent with the high incidence of Staphylococcal infection, which is a known risk factor for embolic complications [8]. Since our study has been conducted in a high-volume surgical center, thereby limiting the referral of uncomplicated IE eligible to medical therapy, the overall percentage of patients undergoing surgery was high. The proportion of operated patients was similar in IDA and non-IDA groups. Of the 45 IDA patients treated surgically, 28 (62%) presented an infection involving left-sided valves. The proportion of left-sided infection in IDA patients treated surgically was higher but not significantly different compared with patients without surgical indication (62 vs 44%).

IDA patients who were treated medically for the absence of surgical indication had better—though non-significantly—survival than those who were operated because of complicated IE. IE relapses during the follow-up were more frequent in IDA than non-IDA patients (28.3 vs. 3.3%, p < 0.001).

**Fig. 1:**
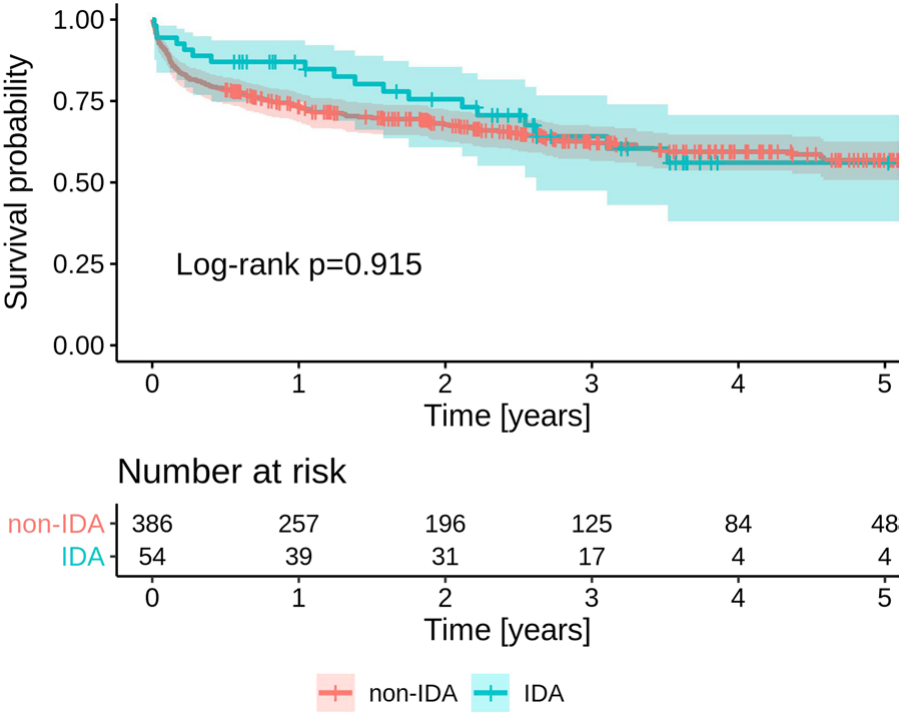
Kaplan–Meier survival analysis of 440 patients with infective endocarditis associated and non-associated with intravenous drug abuse (IDA)

At univariate Kaplan–Meier analysis, IDA was not associated with higher mortality (Fig. 1).

However, as previously reported [10], drug abusers had a more than doubled long-term risk of death at multivariate analysis (HR 2.3, 95%CI 1.1–4.7, p = 0.015), adjusting for age (HR per unit 1.034, 95%CI 1.018–1.050, p < 0.001), EuroSCORE II (HR per unit 1.015, 95%CI 1.008–1.023, p < 0.001), double-valve infection (HR 2.0, 95%CI 1.4–3.0, p < 0.001), and Streptococcus infection (HR 0.5, 95%CI 0.3–0.8, p = 0.009), which were the variable retained in the final model.

Of 54 IDA patients, 19 died during the follow-up; in this subgroup, univariable Kaplan–Meier survival analyses showed no significant differences in mortality by age, gender, associated viral infections (HIV, HBV, HCV), microbiologic yield (including polymicrobial infection), infection site, type of valve affected (prosthetic or native), EuroSCORE II, embolism detected on admission, recurrent IE, nor subsequent IE relapses. The only IDA patient not operated because of refusal despite indication died in hospital. In conclusion, we did not identify any clinical predictor—not even the occurrence of multiple relapses—of the potential unfavourable risk–benefit ratio of surgery in IDA patients.

## Discussion

Our study described the clinical and microbiologic characteristics and the long-term outcome of surgery in a clinical cohort of patients with IDA-associated IE operated in a single centre.

We found a prevalence of 13.4%, stable over the study period, which contrasts with the survey of Rudasill et al. [1] in the United States, where IDA-associated accounted for 22.2% of 96,344 cases of IE, with an increasing incidence between 2010 and 2015. Our data are much closer to the European epidemiology as reported by the EURO-ENDO registry [11], where a history of intravenous drug abuse was present in only 6.9% of patients with IE.

As reported in other studies, IDA-associated IE affected mostly right-sided heart valves [2, 5], polymicrobial flora was common [5], and the most frequent microbiologic agent was Staphylococcus aureus consistent with non-sterile drug injections [5].

In our centre, patients with IDA-associated and non-associated IE were operated in similar proportions. Surgical treatment of drug abusers remains challenging, considering the high rate of relapses and the consequent concern on the potential high risk–benefit ratio of surgery. In this setting, surgical indications include the failure of antibiotic therapy, septic complications, or severe valvular dysfunction [7]. Other series reported that the risk of death or reoperation was ten times higher in IDA-associated than non-associated IE [4]. Rohn et al. [3], over a follow-up exceeding four years, found that survival was significantly impaired in patients with recurrent IE. Weymann et al. [13] identified pre-operative NYHA class grade IV as a risk factor for early mortality.

In our experience, mortality over a 3-year follow-up was similar, at univariate analysis, in IDA-associated and non-associated IE: a finding that likely reflects the younger age and the lower burden of comorbidities of the group of drug abusers. The immediate clinical outcome was good even in patients who needed redo surgery for recurrent IE, and EuroSCORE II did not predict short-term mortality. This suggests that, at least in expert hands, repeated operations can be performed as safely as first surgery, given the overall favourable pre-operative conditions (lower age and fewer comorbidities) of these patients.

On the other hand, the long-term outcome of IDA patients is very unsatisfactory, as shown by Rohn et al. [3] but, at least in large part, likely attributable to continuing drug abuse, as suggested by a 2.3 increase in the risk of death at multivariate analysis in our series.

In contrast with the experience of Rohn et al. [3], we did not find higher mortality even after multiple relapses. Moreover, since we could not identify any predictor of a futile surgery, this reinforces the concept that any patient with a complicated IE deserves surgery without delay, irrespective of its aetiology.

However, only a complete and permanent cessation of intravenous drug use may change the long-term prognosis of patients with IDA-associated IE. Since opioid use disorder is a chronic disease with a good prognosis when patients are offered evidence-based interventions [14, 15], we should focus on addiction treatment and social support starting early at hospital discharge to improve their long-term prognosis.

The most ethically challenging situation we have experienced is when patients with IDA assume opioids during hospitalization while awaiting surgery: an event that disheartens the whole medical staff, briskly facing the certainty of future relapse. The only possible answer to this problem is to begin early an addiction treatment plan, which must be regarded as an essential component of the whole medical management. Although early as compared to delayed surgery for IE has a better prognosis [8], postponing the operation to achieve the minimum target of short-term abstinence might be worth in drug-addicted patients without an urgent indication for surgery. The prolonged daily antibiotic therapy needed after surgery, usually performed in a day hospital setting, could be the chance to begin strict psychiatric support.

## Conclusions

IDA-associated IE represents a considerable proportion of overall cases of IE, mainly affecting young people. Staphylococcus aureus is the most common microbiologic agent. This subset of IE is not associated with higher short-term mortality, but drug abuse is an independent predictor of long-term prognosis. Considering that patients with uncomplicated IE treated medically have a favourable prognosis, we should reserve surgery for those with a strict indication. On the other hand, since there are no predictors of the adverse risk–benefit ratio of surgery, all patients with a complicated IE should undergo surgery without delay. The main determinant of long-term prognosis in IDA-associated IE is drug abuse itself. Thus, increased focus on addiction treatment starting early during hospitalization is mandatory to improve long-term survival.

## Data Availability

The datasets used and/or analysed during the current study are available from the corresponding author on reasonable request.

## Abbreviations

IDA: Intravenous drug abuse
IE: Infective endocarditis
SD: Standard deviation
IQR: Interquartile range
CKD: Chronic kidney disease
eGFR: Estimated glomerular filtration rate
HR: Hazard ratio
CI: Confidence interval
HIV: Human immunodeficiency virus
HBV: Hepatitis B virus
HCV: Hepatitis C virus
